# Oxidative stress status and reproductive performance of sows during gestation and lactation under different thermal environments

**DOI:** 10.5713/ajas.19.0334

**Published:** 2019-10-21

**Authors:** Yan Zhao, Sung Woo Kim

**Affiliations:** 1 Department of Animal Science and Technology, Shanxi Agricultural University, Taigu, Shanxi 030801, China; 2 Department of Animal Science, North Carolina State University, Raleigh, NC 27606, USA

**Keywords:** Gestation, High Thermal Environment, Lactation, Oxidative Stress, Sow

## Abstract

**Objective:**

Two experiments were conducted using 28 healthy multiparous sows to evaluate the oxidative stress status and reproductive performance of sows during gestation and lactation under different thermal environments.

**Methods:**

Fourteen multiparous sows were used in Exp. 1 under a high thermal environment, and the other 14 multiparous sows were used in Exp. 2 under a moderate thermal environment. In both experiments, reproductive performances of sows were recorded. Plasma samples were collected on d 35, 60, 90, and 109 of gestation, and d 1 and 18 of lactation for malondialdehyde, protein carbonyls, 8-hydroxy-deoxyguanosine, immunoglobulin g (IgG), and IgM analysis.

**Results:**

For sows in Exp. 1, plasma malondialdehyde concentration on d 109 of gestation tended to be greater (p<0.05) than it on d 18 of lactation. Plasma concentration of protein carbonyl on d 109 of gestation was the greatest (p<0.05) compared with all the other days. Plasma concentrations of 8-hydroxy-deoxyguanosine on d 109 of gestation was greater (p< 0.05) than d 18 of lactation in Exp. 1. For sows in Exp. 2, there was no difference of malondialdehyde and protein carbonyl concentration during gestation and lactation. In both Exp. 1 and 2, litter size and litter weight were found to be negatively correlated with oxidative stress indicators.

**Conclusion:**

Sows under a high thermal environment had increased oxidative stress during late gestation indicating that increased oxidative damage to lipid, protein, and DNA could be one of the contributing factors for reduced reproductive performance of sows in this environment. This study indicates the importance of providing a moderate thermal environment to gestating and lactating sows to minimize the increase of oxidative stress during late gestation which can impair reproductive outcomes.

## INTRODUCTION

Reactive oxygen species (ROS) including superoxide anion (O_2_^•−^), hydroxyl radical (^•^OH), and hydrogen peroxide (H_2_O_2_) are produced during biochemical processes within an animal’s body [1]. If the balance between the production of ROS and antioxidant defense is disturbed, the animal can suffer from oxidative stress. Accumulation of ROS could react with biological macromolecules such as proteins, lipids, and nucleic acids, resulting in lipid peroxidation, protein damage, and DNA damage [2,3]. In sows, external factors such as social stress and environmental stressors can lead to increased oxidative stress [4,5]. Zhao et al [5] studied the oxidative stress status of gestating and lactating sows under different gestational housing systems and social ranks. The results showed that sows under intense social stress could increase oxidative stress level during gestation and lactation [5]. Bottje et al [4] showed that environmental stressors such as high dust and ammonia levels in poultry house environment can cause oxidative stress in lung lining fluid of broilers.

Heat stress is another important environmental stressor to animal’s reproductive performance especially during summer months in tropical and subtropical regions of the world. It has a significant negative impact on reproductive performance of sows including impaired embryonic development [6], reduced feed intake and milk production in sows [7,8], and delayed puberty of gilts [9], which result in summer infertility and productivity losses that amount to millions of dollars. In the last decades, genetic improvement of sows for high reproductive performance results in increased metabolic heat production which makes sows more susceptible to the high thermal environment [10,11]. Previous studies found that sows were under severe catabolic status during late gestation and lactation [12,13], and their oxidative stress levels increased during the same period [14]. However, the negative impact of high thermal environment on sow’s oxidative stress status have not been well studied, and it is not known if oxidative stress status is related to reproductive performance of sows under different thermal environments. Therefore, we hypothesized that oxidative stress status of sows could elevate during late gestation and lactation when sows were housed under high thermal environment, and the elevated oxidative stress could be detrimental to the reproductive performance of sows. Thus, the objective of this study was to investigate oxidative stress status and its relationship with reproductive performance of sows during gestation and lactation under moderate and high thermal environments.

## MATERIALS AND METHODS

### Animal care

Procedures used in this study were reviewed and approved by the North Carolina State University Animal Care and Use Committee.

### Animals and experimental design

**Figure 1:**
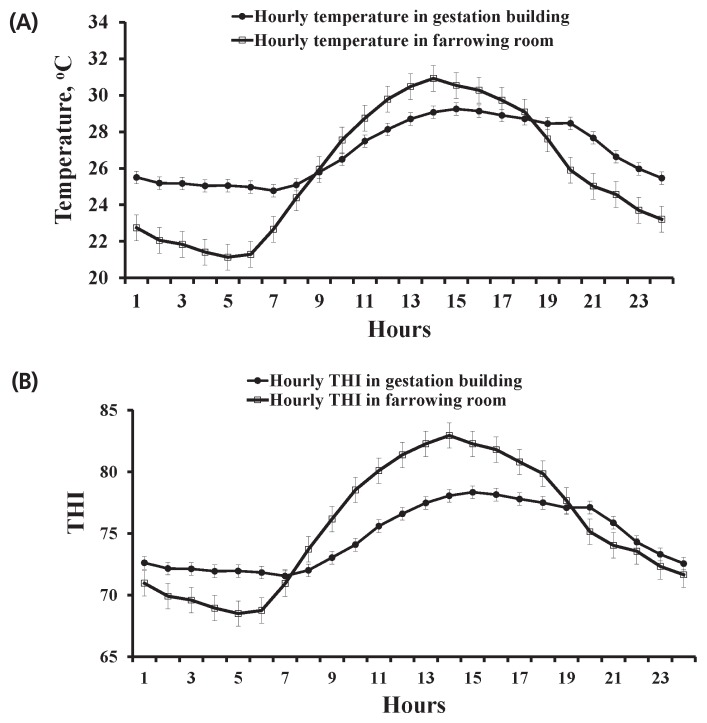
Average hourly temperature (A) and temperature-humidity index (THI) (B) in a day from June to August for sows in a high thermal environment in Exp. 1. Average daily minimum and maximum temperatures of 24.8°C±2.2°C, 30.3°C±2.9°C in gestation building, and 22.1°C±1.8°C, 30.9°C±2.6°C in farrowing building, respectively.

**Figure 2:**
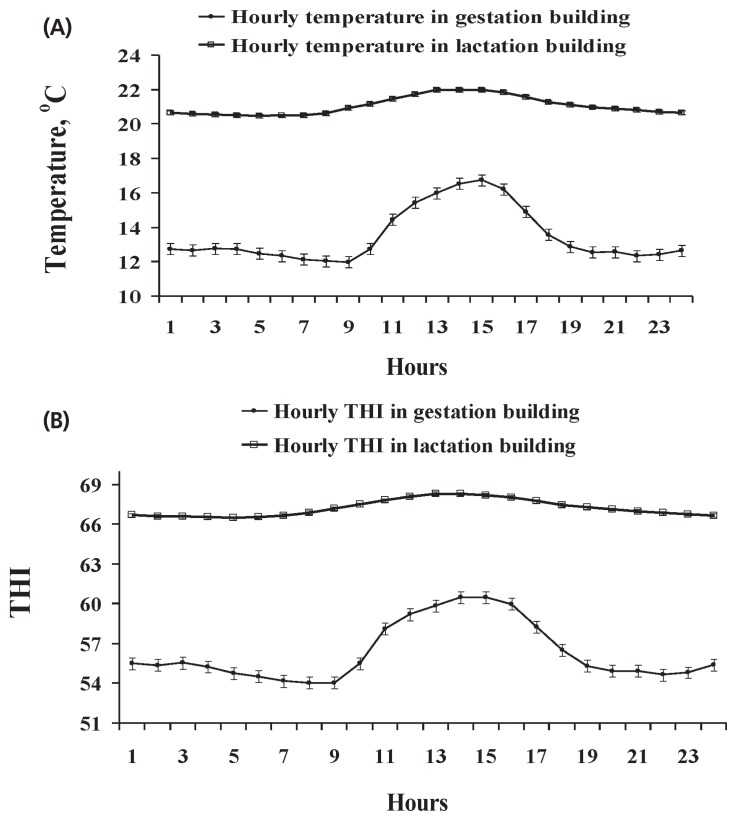
Average hourly temperature (A) and temperature-humidity index (THI) (B) in a day from November to January for sows in a moderate thermal environment in Exp. 2. Average daily minimum and maximum temperatures of 11.9°C±3.0°C, 16.7°C±3.5°C in gestation building, and 20.4°C±2.1°C, 22.3°C±2.0°C in farrowing building, respectively.

Two experiments were carried out using 28 healthy multiparous sows at the North Carolina State University Swine Educational Unit (Raleigh, NC, USA), in the summer (Exp. 1, June to August with average daily maximum and minimum temperature of 30.3°C±2.9°C and 24.8°C±2.2°C in the gestation building, and 30.9°C±2.6°C and 22.1°C±1.8°C in the farrowing building, respectively) and winter (Exp. 2, November to January with the average daily maximum and minimum temperature of 16.7°C±°C and 11.9°C±3.0°C in the gestation building, and 22.3°C±2.0°C and 20.4°C±2.1°C in the farrowing building, respectively). In Exp. 1, 14 multiparous sows (initial body weight [BW], 245.7±38.7 kg; parity, 5.8±3.2; Landrace×Large White) were selected and assigned to a high thermal environment on d 35 of gestation. In Exp. 2, the other group of 14 multiparous sows (initial BW, 243.2± 30.3 kg; parity, 5.1±1.3; Landrace×Large White) were selected and assigned to a moderate thermal environment on d 35 of gestation. Sows from both experiments were housed in the same gestation and farrowing buildings and managed with standard procedures. A negative pressure system was used to ventilate the gestation building with four 1,100-rpm, 61-cm wall fans. The farrowing building had an evaporative cooling system and two 1,100-rpm, 61-cm wall fans. Water flow through the evaporative cooling system was controlled by an on-off thermostat which was set at 25°C, and one gas heater was provided during winter season in the farrowing room. The thermal environment inside the facilities was monitored hourly using data loggers (Tinytag Plus 2, TPG-4500, Gemini Data Loggers, Chichester, UK) to measure temperature and relative humidity. Average hourly ambient temperature and temperature-humidity index (THI) within a day in the gestation and farrowing buildings in Exp. 1 and 2 were showed in Figure 1 and 2, respectively. In Exp. 1, sows were exposed to an ambient temperature above 25°C for an average of 17 h and 14 h per day in gestation building and farrowing building, respectively (Figure 1A). The hourly THI was ranged from 72 to 78 in the gestation building and from 69 to 83 in the farrowing building (Figure 1B). On the other hand, sows in Exp. 2 were kept in a moderate thermal environment as shown in Figure 2. The hourly ambient temperature was from 12°C to 17°C in the gestation building and 19°C to 21°C in the farrowing building (Figure 2A). The hourly THI was ranged from 54 to 61 in the gestation building and from 65 to 67 in the farrowing building (Figure 2B).

The estrus of weaning sows in these 2 experiments was detected daily by the immobilization response as a reaction to a heat check boar after 3 d back to the breeding barn. Sows were artificially inseminated twice (12 h apart) after estrus onset. Each insemination was conducted within 18 h of semen collection (Duroc semen). Pregnancy was detected and confirmed at d 30 post-breeding using an ultrasound scanner (VSS700 EZ Preg Checker, Veterinary Sales & Service Inc., Stuart, FL, USA). In the gestation building, sows were individually housed in gestation crates (2.0×0.64 m) with semi-automatic feeders, individual drinkers, and slatted flooring. They were fed 2.2 kg gestation diet daily which contained 13.3% crude protein (CP) and 3.3 Mcal metabolizable energy (ME)/kg (Table 1). Water was *ad libitum* during gestation and lactation. On d 108 of gestation, all of the sows were weighed and moved to farrowing crates (2.1×1.5 m) in an adjacent farrowing building. Three sows in Exp. 1 and 2 sows in Exp 2. did not maintain their pregnancy and did not farrow. During lactation, sows were fed *ad libitum* by electronic feeders (JYGA Technologies, Saint-Nicolas, QC, Canada) in the farrowing building. Lactation diet contained 15.8% CP and 3.5 Mcal ME/kg (Table 1). The gestation and lactation diets were formulated to meet or exceed NRC nutrient requirements [15], and formulations were the same in Exp. 1 and 2. In both experiments, any feed left from the previous day was removed and weighed around 0800 h, and the difference between the amount of feed provided and remained was used as an estimate of daily feed intake. The BW of sows was measured on d 35 and 109 of gestation, and d 1 and 18 of lactation. Backfat thickness (10th rib, 6 cm off-midline) of each sow was measured on d 1 and 18 of lactation using an ultrasound scanner (VSS700 EZ Preg Checker, Veterinary Sales & Service Inc., USA). After farrowing, individual weight of piglet and litter size of sows on d 1 (total number of piglets, born alive, stillborn, and mummy) and 18 of lactation were measured.

### Blood sampling

In both Exp. 1 and 2, blood was sampled via jugular venipuncture from all of the sows restrained by snout snare between 0900 and 1000 h on d 35, 60, 90, and 109 of gestation in the gestation building, and d 1 and 18 of lactation in the farrowing building. Blood was collected using 9 mL ethylenediaminetetraacetic acid-coated syringes and disposable 16-gauge×0.1-mm hypodermic needles (MONOVETTE, Sarstedt, Newton, NC, USA; Air-Tite Product Co. Inc., Virginia Beach, VA, USA). Plasma samples were obtained by centrifugation (5810 R, Eppendorf AG, Hamburg, Germany) at 3,000 g, 15 min, 4°C, then allocated into 1.5 mL microcentrifuge tubes, kept in liquid nitrogen for 1 h, and stored at −80°C until analysis.

### Analysis of oxidative stress indicators

Plasma samples from Exp. 1 and 2 were used to measure concentrations of malondialdehyde (MDA), protein carbonyl, and 8-hydroxy-deoxyguanosine (8-OHdG). Concentrations of MDA were measured using the thiobarbituric acid reactive substances assay kit (Cell Biolabs, San Diego, CA, USA) according to the method described by Zhao et al [5]. Plasma samples and MDA standards were incubated and reacted with thiobarbituric acid at 95°C. After centrifuge and butanol extraction, samples and standards were read at 532 nm by a spectrophotometric plate reader (Synergy HT, BioTek Instruments, Winooski, VT, USA). Samples were quantified against the standard curve which was constructed by MDA standards. The detective limit of MDA analysis was 0.98 μM.

Protein concentration in each plasma sample was measured by a bicinchoninic acid protein assay kit (Pierce Biotechnology, Rockford, IL, USA). Then all plasma samples were diluted with bicinchoninic acid to reach protein concentration at 10 μg/mL before measuring protein carbonyl. Protein carbonyl concentration in each diluted plasma sample was measured via the protein carbonyl ELISA kit (Cell Biolabs, USA) according to the method described by Shen et al [16]. The protein carbonyl presented in the sample or standard was first derivatized to dinitrophenyl hydrazine and probed with an anti-dinitrophenyl antibody, then incubated with a secondary antibody. Finally substrates and stop solutions were added. Standards and samples were read at 450 nm by a spectrophotometric plate reader (Synergy HT, BioTek Instruments, USA). Protein carbonyl concentrations in samples were quantified against the standard curve which was drawn by protein carbonyl standards. The detective limit for protein carbonyl was 0.375 nmol/mg.

Concentrations of 8-OHdG in plasma samples were measured using the oxidative DNA damage enzyme-linked immunosorbent assay (ELISA) kit (Cell Biolabs, USA) according to the method described by Weaver and Kim [17]. Briefly, plasma samples and 8-OHdG standards were first added into a 96-well plate. Then an anti-8-OHdG monoclonal antibody was added, followed by adding a secondary antibody. A substrate solution and stop solution were added. The absorbance of each well was read at 450 nm by a spectrophotometric plate reader (Synergy HT, BioTek Instruments, USA). Concentrations of 8-OHdG in plasma samples were quantified against the standard curve which was constructed by 8-OHdG standards. The detective limit for 8-OHdG was 0.078 ng/mL.

### Immunoglobulin evaluation

In both experiments, concentrations of immunoglobulin G (IgG) and IgM in sows’ plasma on d 109 of gestation, d 1 and 18 of lactation were measured by ELISA kits (Bethyl, Montgomery, TX, USA) according to the method described by Chaytor et al [18]. Briefly, goat anti-pig IgG or IgM were used as capture antibodies to coat wells. All of the samples were diluted to 1:100,000 for IgG and IgM measurement. Horseradish peroxidase goat anti-pig IgG or IgM was used as the detection. The plate was read at 450 nm by an ELISA plate reader (Synergy HT, BioTek Instruments, USA) and software (KC4 Data Analysis Software, BioTek Tnstrument, USA). Sample concentration was quantified against the standard curve which was drawn by standards. Detective limits were 7.8 ng/mL for IgG, and 15.6 ng/mL for IgM, respectively.

### Statistical analysis

Exp. 1 and 2 were completely randomized designs. Data from oxidative stress indicators and immunoglobulin assay were compared among different gestation and lactation days in both experiments using the MIXED procedure of SAS (SAS Inst. Inc., Cary, NC, USA). Oxidative stress data from d 35 of gestation was used as a covariance when analyzing the oxidative stress indicators. The individual sow was the experimental unit. Day was a fixed effect and the sow was a random effect. The canonical correlation between reproductive performance and oxidative stress indicators on different days in Exp. 1 and 2 were analyzed by the CANCORR procedure of SAS (SAS Inst., Inc., USA), respectively. Data was considered statistically different when probability values were less than 0.05, and probability less than 0.1 and equal or greater than 0.05 was considered as a trend.

## RESULTS

### Oxidative stress parameters

Plasma concentrations of MDA, protein carbonyl, and 8-OHdG were compared among days during gestation and lactation in Exp. 1 and 2, respectively (Table 2). For sows in Exp. 1, plasma MDA concentration on d 109 of gestation tended to be greater (p = 0.096) than it on d 18 of lactation (Table 2). Plasma concentration of protein carbonyl on d 109 of gestation was the greatest (p<0.05) compared with all the other days (Table 2). Besides, protein carbonyl concentration on d 18 of lactation was greater (p<0.05) than concentrations on d 60 and 90 of gestation. Plasma concentrations of 8-OHdG on d 109 of gestation was greater (p<0.05) than d 18 of lactation in Exp. 1 (Table 2). For sows in Exp. 2, there was no difference of MDA concentration among gestation and lactation days (Table 2). Protein carbonyl concentration did not differ among days (Table 2). However, plasma concentration of 8-OHdG on d 18 of lactation was smaller (p<0.05) than all the other days in Exp. 2 (Table 2).

### Reproductive performance of sows

In Exp. 1, sows gained 35.0 kg from d 35 to 109 of gestation, but lost 1.5 kg from d 1 to 18 of lactation. Sows consumed 4.3±1.1 kg feed daily during lactation. Sows had 9.5±2.9 pigs born alive per litter, and weaned 7.4±2.1 pigs per litter at the end of lactation. Litters gained 27.1±8.7 kg from d 1 to 18 of lactation. Piglets gained 204.4±41.3 g daily during lactation (Table 3).

In Exp. 2, sows gained 46.6 kg from d 35 to 109 of gestation, but lost 14.7 kg from d 1 to 18 of lactation. Sows consumed 4.6±1.1 kg feed daily during lactation. Sows had 11.9±1.8 pigs born alive per litter, and weaned 10.4±1.2 pigs per litter at the end of lactation. Litters gained 37.5±9.5 kg from d 1 to 18 of lactation. Piglets gained 199.5±41.2 g daily during lactation (Table 3).

### Immunoglobulin evaluation

Plasma concentrations of IgG and IgM were compared among d 109 of gestation, and d 1 and 18 of lactation in Exp. 1 and 2, respectively (Table 4). For sows in Exp. 1, there was no difference for plasma concentrations of IgG and IgM on d 109 of gestation, and d 1 and 18 of lactation (Table 4). For sows in Exp. 2, plasma concentration of IgM did not differ among different days. However, plasma concentration of IgG on d 18 of lactation was greater (p<0.05) than it on d 1 of lactation (Table 4).

### Canonical correlation analysis between oxidative stress and reproductive performance

Three sets of canonical relationships between oxidative stress indicators and reproductive performance of sows were obtained in Exp. 1 (Table 5). The first set of variables are oxidative stress indicators including concentrations of MDA, protein carbonyl, and 8-OHdG on d 60 and 90 of gestation, and d18 of lactation (*M*_d60_, *P*_d60_, *O*_d60_, *M*_d90_, *P*_d90_, *O*_d90_, *M*_d18_, *P*_d18_, and *O*_d18_). The second set of variables are BW and backfat of sows, and piglet weight on d 3 and 18 of lactation (*X*_1_, *X*_2_, *X*_3_, *X*_4_, *X*_5_, and *X*_6_). The correlation coefficients in the first set of canonical relationship reached a significant level (p<0.05), and the other two sets showed tendency (p = 0.093 and 0.059), suggesting that there is a significant canonical correlation between oxidative stress and reproductive performance of sow in Exp. 1 (Table 5). From the linear expression of canonical variable composition in the first set of canonical relationship, *M*_d60_, *O*_d60_, and *X*_2_ were relatively large (Table 5), showing that plasma concentration of MDA and 8-OHdG on d 60 of gestation are negatively correlated with BW of sows on d 18 of lactation. As can be seen from the linear expression of canonical variables in the second set of canonical relationship, *M*_d90_, *P*_d90_, and *X*_4_ were relatively large (Table 5), showing that plasma concentration of MDA and protein carbonyl on d 90 of gestation are negatively correlated with backfat of sows on d 18 of lactation. In the third set of canonical relationship, there was a large load on *M*_d18_, *P*_d18_, and *X*_6_ (Table 5), suggesting that plasma concentration of MDA and protein carbonyl on d 18 of lactation are negatively correlated with piglet weight on d 18 of lactation.

Two sets of canonical relationships between oxidative stress indicators and reproductive performance of sows in Exp. 2 and the composition of canonical variables are shown in Table 6. The first set of variables are oxidative stress indicators including concentrations of MDA, protein carbonyl, and 8-OHdG on d 90 of gestation, and d3 of lactation (*M*_d90_, *P*_d90_, *O*_d90_, *M*_d3_, *P*_d3_, and *O*_d3_). The second set of variables are piglet weight on d 3 and 18 of lactation, litter size of born alive and wean of sows (*X*_5_, *X*_6_, *X*_7_, and *X*_8_). The correlation coefficients in the first and second set of canonical relationships reached a significant level (p<0.05), suggesting that there is a significant canonical correlation between oxidative stress and reproductive performance of sow in Exp. 2 (Table 6). From the linear expression of canonical variable composition in the first set of canonical relationship, *M*_d3_, *O*_d3_, and *X*_6_ were relatively large (Table 6), showing that MDA and 8-OHdG on d 3 of lactation are negatively correlated with piglet weight on d 18 of lactation. As can be seen from the linear expression of canonical variables in the second set of canonical relationship, there was a large load on *P*_d90_ and *X*_8_ (Table 6), suggesting that protein carbonyl on d 90 of gestation is negatively correlated with litter size of sows on d 18 of lactation.

## DISCUSSION

In this study we have confirmed that sows under a high thermal environment had increased oxidative stress during late gestation, and the increased oxidative damage to lipid, protein, and DNA could be one of the contributing factors for reduced reproductive performance of sows under the high thermal environment. Hence, we accepted the hypothesis that oxidative stress status of sows could elevate during late gestation and lactation when sows are housed under high thermal environment, and the elevated oxidative stress could be detrimental to the reproductive performance of sows.

In this study, sows in Exp. 1 were under heat stress condi tion indicated by the observed ambient temperatures and THI. We observed that the ambient temperature in Exp. 1 were above 25°C for an average of 17 h/d in the gestation building and 14 h/d in the farrowing building. Environmental temperature remaining above 25°C could cause heat stress to sows [19]. Meanwhile, high levels of THI (72 to 78 in the gestation building, and 69 to 83 in the farrowing building) was also observed in Exp. 1. The THI is a combination of temperature and humidity which has been used to indicate heat stress environment. It was reported that the THI value less than 74 was classified as a safe, whereas critical when greater than 74 in sows [20]. The observed high THI in Exp. 1 can be detrimental to sows, because the high temperature and high humidity together can limit their heat loss through evaporation [21]. These data indicated that sows in Exp. 1 were under heat stress condition. On the other hand, based on the average daily maximum and minimum temperatures and THI recorded in Exp. 2, it seems that sows in Exp. 2 were kept within their thermos-neutral environment.

Our Exp. 1 was conducted from June to August in North Carolina, which we observed the reduced reproductive performance of sows indicated by the less number of total born, born alive, more still born piglets, and small litter size and litter weight gain. These results indicated that the reproductive performance of sows in Exp. 1 was negatively affected by the heat stress environment. The smaller litter size of sow in Exp. 1 contributed to reduce mobilizing of their body reserve for less milk production, which may explain why sows in Exp. 1 lost less BW and backfat during lactation compared with sows in Exp. 2. Similar result was reported that the over-all reproductive performance of sows reduced in large confinement units in North Carolina during hot months from June to October [22].These findings were also consistent with other studies which showed that sows under high thermal environment during gestation farrowed fewer born alive with increased number of stillborn [8]. On the other hand, feed intake of lactating sows was not the main factor for the reduced milk production in Exp.1. Because feed intake in Exp. 1 was similar to that in Exp. 2, and it did not show significant reduction as some other studies have found [23]. This may be because it took a few days for sows to adjust the electronic feeders in the farrowing room.

Besides the reduced reproductive performance of sows in Exp. 1, heat stress environment also affected the oxidative stress status of sows indicated by the enhanced oxidative damage to lipid, protein, and DNA during late gestation. The plasma MDA, protein carbonyl, and 8-OHdG concentration increased during late gestation which showing that sows could suffer severe oxidative damage during late gestation under heat stress environment. The current results indicate that oxidative stress could be one of stress responses caused by high thermal environment. On the other hand, sow in Exp. 2 did not show the similar pattern with Exp. 1. Although sows had increased oxidative DNA damage during gestation and early lactation compared with late lactation, other oxidative indicators for protein and lipid did not enhanced, indicating that sows in Exp. 2 did not suffer severe oxidative stress during gestation and lactation when they were housed under moderate thermal environment. Previous studies showed that sows were under severe catabolic status during late gestation and lactation which could induce oxidative damage and decrease antioxidants concentration at the same period [12–14]. Studies also found that the ROS production increased under the high temperature treatment [24,25], which could explain why sows in Exp. 1 showed enhanced oxidative stress. However, the molecular mechanisms responsible for excessive ROS production or lowered antioxidant defenses under heat stress are still not known in sows. There are some reports in poultry which found that heat stressed chickens exhibit overproduction of mitochondrial ROS in skeletal muscle, which might result from enhanced substrate oxidation and downregulation of avian uncoupling protein [26,27].

As discussed above, heat stress environment negatively affected the reproductive performance and enhanced the oxidative damage to sows during late gestation and lactation. We hypothesized that the elevated oxidative stress could be detrimental to the reproductive performance of sows. Therefore, we investigated the relationship between oxidative stress indicators and reproductive performance of sows during gestation and lactation under both moderate and high thermal environments. The canonical correlation coefficients were close to 1 for all the sets of canonical relationships, and the linear expression of canonical variable compositions showed that there were negative correlations between oxidative stress indicators and piglet weight, BW, backfat, and litter size of sows in current studies. These results indicates a strong correlation between oxidative stress indicators and reproductive performance of sows. Similar results were reported in previous study, which showed that oxidative damages to lipid, protein, and DNA were negatively correlated with reproductive performance of gestating sows under social stress [5]. Excessive ROS could negatively affect multiple physiological processes from oocyte maturation to fertilization, embryo development and pregnancy [28]. Study on pregnant women showed that oxidative stress can increase membrane damage which is related with fetal growth retardation and cause a higher risk of prenatal mortality [29].

In Exp. 1, plasma concentrations of IgG and IgM were not different among days, indicating that sows did not suffer immune challenge during the study. The plasma concentration of IgG on d 1 of lactation in Exp. 2 was smaller than it on d 18 of lactation, but both of them were within the normal range [30]. The decreased plasma concentration of IgG on d 1 of lactation may be because more IgG in the blood were secreted into the colostrum.

## CONCLUSION

Taken together, sows under a high thermal environment had increased oxidative stress during late gestation. These oxidative stress indicators were negatively correlated with reproductive performance of sows, suggesting that increased oxidative damage to lipid, protein, and DNA could be one of the contributing factors for reduced reproductive performance of sows under the high thermal environment. This study indicates the importance of providing a moderate thermal environment to gestating and lactating sows to minimize the increase of oxidative stress during late gestation which can impair reproductive outcomes. Our findings in this study also shows the importance for future studies to regulate oxidative stress status of sow during gestation and lactation under heat stress.

## Figures and Tables

**Table 1:** Composition of gestation and lactation diets (as-fed basis) in Exp. 1 and 2

Item	Gestation	Lactation
Ingredient		
Corn, yellow (%)	81.30	74.00
Soybean meal, 48% CP (%)	13.85	19.60
Poultry fat (%)	1.00	3.99
L-Lys (%)	0.00	0.25
L-Thr (%)	0.00	0.01
Limestone (%)	1.11	1.08
Dicalcium phosphate (%)	2.05	2.38
Salt (%)	0.50	0.50
Trace mineral permix1) (%)	0.15	0.15
Vitamin permix2) (%)	0.04	0.04
Total	100.00	100.00
Calculated composition		
DM (%)	89.7	90.1
ME (Mcal/kg)	3.3	3.5
CP (%)	13.3	15.8
Lys (%)	0.63	0.92
Met (%)	0.49	0.51
Trp (%)	0.14	0.17
Thr (%)	0.49	0.55
Ca (%)	1.03	1.12
Total P (%)	0.69	0.76

CP, crude protein; DM, dry matter; ME, metabolizable energy.

1) The trace mineral premix provided per kilogram of complete diet: 3.96 mg of Mn as manganous oxide; 16.5 mg of Fe as ferrous sulfate; 16.5 mg of Zn as zinc sulfate; 1.65 mg of Cu as copper sulfate; 0.30 mg of I as ethylenediamine dihydroiodide; and 0.30 mg of Se as sodium selenite.

2) The vitamin premix provided per kilogram of complete diet: 8,228 IU of vitamin A as vitamin A acetate; 1,173 IU of vitamin D_3_; 47 IU of vitamin E; 0.03 mg of vitamin B_12_; 5.88 mg of riboflavin; 23.52 mg of D-pantothenic acid as calcium panthonate; 35.27 mg of niacin; 0.24 mg of biotin; 1.76 mg folic acid; 3.88 mg menadione.

**Table 2:** Oxidative stress indicators of sows under a high thermal environment (HT)1) in Exp. 1 and a moderate thermal environment (MT)1) in Exp. 2

Item	Gestation				Lactation		SEM	p-value
	
	d 352)	d 60	d 90	d 109	d 1	d 18		
Exp. 1								
n3)	11	11	11	11	11	11		
Malondialdehyde (μM)	6.14	6.39^AB^	7.51^AB^	8.54^A^	8.39^AB^	6.02^B^	1.05	0.096
Protein carbonyl (nmol/mg)	1.41	1.00^a^	1.06^a^	2.29^c^	1.40^ab^	1.76^b^	0.13	<0.001
8-hydroxy-deoxyguanosine (ng/mL)	0.32	0.59^ab^	0.61^ab^	0.95^b^	0.62^ab^	0.29^a^	0.12	0.006
Exp. 2								
n3)	12	12	12	12	12	12		
Malondialdehyde (μM)	4.42	4.63	4.00	2.56	3.53	5.26	0.98	0.285
Protein carbonyl (nmol/mg)	1.59	1.50	0.72	1.03	1.29	0.76	0.22	0.122
8-hydroxy-deoxyguanosine (ng/mL)	0.71	1.00^b^	0.90^b^	1.07^b^	1.04^b^	0.48^a^	0.12	<0.001

SEM, standard error of the mean.

1) HT, high thermal environment (average daily minimum and maximum temperatures of 24.8°C±2.2°C, 30.3°C± 2.9°C in gestation building, and 22.1°C±1.8°C, 30.9°C±2.6°C in farrowing building, respectively); MT, moderate thermal environment (average daily minimum and maximum temperatures of 11.9°C±3.0°C, 16.7°C±3.5°C in gestation building, and 20.4°C±2.1°C, 22.3°C±2.0°C in farrowing building, respectively).

2) Oxidative stress data from d 35 of gestation was used as a covariance when analyzing the oxidative stress indicators.

3) Initial number of sows was 14. There were 3 sows in Exp. 1 and 2 sows in Exp. 2 did not maintain pregnancy and did not farrow which were excluded from the study.

a–c Means within a row with different superscripts differ (p<0.05).

A,B Means within a row tend to differ (0.05≤p<0.10).

**Table 3:** Reproductive performance of sows under a high thermal environment (HT)1) in Exp. 1 and a moderate thermal environment (MT)1) in Exp. 2

Item	Exp. 1	SD2)	Exp. 2	SD2)
n3)	11		12	
Parity	5.8	3.2	5.1	1.3
Body weight of sows (kg)				
d 35 of gestation	246	39	243	30
d 109 of gestation	281	35	290	27
d 1 of lactation	275	32	276	28
d 18 of lactation	273	29	261	31
Body weight changes (kg)				
Gestation (d 35 to 109)	35	17	47	14
Lactation (d 1 to 18)	−5	13	−13	17
Backfat of sows4) (mm)				
d 1 of lactation	17.0	2.5	15.8	3.9
d 18 of lactation	16.6	2.9	14.4	3.8
Change from d 1 to 185)	−0.4	2.7	−1.4	1.6
ADFI of sows (kg)	4.3	1.1	4.6	1.1
Litter size (pig)				
d 1, total born	12.5	2.9	13.6	2.2
d 1, born alive	9.5	2.9	11.9	1.8
d 1, stillborn	2.5	1.9	1.2	1.2
d 1, mummy	0.5	0.8	0.4	0.9
d 18	7.4	2.1	10.4	1.2
Change from d 1 to 185)	−2.2	1.7	−1.5	1.7
Litter weight (kg)				
d 16)	15.0	3.7	17.9	2.9
d 18	42.1	11.7	55.4	11.2
Gain from d 1 to 185)	27.1	8.7	37.5	9.5
Piglet weight (kg)				
d 17)	1.66	0.42	1.52	0.20
d 18	5.78	0.96	5.42	0.78
ADG from d 1 to 18 (g/d)	204	41	200	41

SD, standard deviation; ADFI, average daily feed intake; ADG, average daily gain.

1) HT, high thermal environment (average daily minimum and maximum temperatures of 24.8°C±2.2°C, 30.3°C±2.9°C in gestation building, and 22.1°C±1.8°C, 30.9°C±2.6°C in farrowing building, respectively); MT, moderate thermal environment (average daily minimum and maximum temperatures of 11.9°C±3.0°C, 16.7°C±3.5°C in gestation building, and 20.4°C±2.1°C, 22.3°C±2.0°C in farrowing building, respectively).

2) Results are expressed as mean with SD.

3) Initial number of sows was 14 for both Exp. 1 and 2. There were 3 sows in Exp. and 2 sows in Exp. 2 did not maintain pregnancy and did not farrow which were excluded from the study.

4) Measured at the P2 position (locate at left side of the10th rib, and 6 cm away from the spine).

5) Difference between d 1 and 18 of lactation

6) Litter weight at birth includes piglets born alive only.

7) Weight of piglets born alive.

**Table 4:** Immunological parameters of sows under a high thermal environment (HT)1) in Exp. 1 and a moderate thermal environment (MT)1) in Exp. 2

Item	Plasma			SEM	p value

	d 109 of gestation	d 1 of lactation	d 18 of lactation		
Exp. 1					
n2)	11	11	11	-	-
IgM (mg/mL)	3.25	2.55	2.33	0.41	0.419
IgG (mg/mL)	19.38	17.98	19.67	1.69	0.898
Exp. 2					
n2)	12	12	12	-	-
IgM (mg/mL)	2.96	2.36	2.53	0.28	0.621
IgG (mg/mL)	19.20^ab^	16.22^a^	20.07^b^	1.19	0.031

SEM, standard error of the mean; IgM, immunoglobulin M; IgG, immunoglobulin G.

1) HT, high thermal environment (average daily minimum and maximum temperatures of 24.8°C±2.2°C, 30.3°C± 2.9°C in gestation building, and 22.1°C±1.8°C, 30.9°C±2.6°C in farrowing building, respectively); MT, moderate thermal environment (average daily minimum and maximum temperatures of 11.9°C±3.0°C, 16.7°C±3.5°C in gestation building, and 20.4°C±2.1°C, 22.3°C±2.0°C in farrowing building, respectively).

2) Initial number of sows was 14. There were 3 sows in Exp. 1 and 2 sows in Exp. 2 did not maintain pregnancy and did not farrow which were excluded from the study.

a,b Means within a row with different superscripts differ (p<0.05).

**Table 5:** Canonical correlations between oxidative stress indicators and reproductive performance of sows in Exp. 1

The first set of variables1)	The second set of variables2)	Canonical correlation coefficient	p-value	Canonical variable composition
Oxidative stress indicators	Sow BW	0.990	0.023	*V*_1_ = 0.924*M*_d60_ + 0.340*P*_d60_ + 0.652*O*_d60_ *W*_1_ = 0.364*X*_1_ − 1.222*X*_2_
Oxidative stress indicators	Sow backfat	0.879	0.093	*V*_1_ = 1.211*M*_d90_ + 1.143*P*_d90_ + 0.621*O*_d90_ *W*_1_ = 0.277*X*_3_ − 1.026*X*_4_
Oxidative stress indicators	Piglet weight	0.960	0.059	*V*_1_ = −0.988*M*_d18_ − 1.448*P*_d18_ − 0.413*O*_d18_ *W*_1_ = 0.269*X*_5_ + 0.821*X*_6_

BW, body weight.

1) The first set of variables are oxidative stress indicators including concentrations of malondialdehyde, protein carbonyl, and 8-hydroxy-deoxyguanosine on d 60 and 90 of gestation, and d18 of lactation (*M*_d60_, *P*_d60_, *O*_d60_, *M*_d90_, *P*_d90_, *O*_d90_, *M*_d18_, *P*_d18_, and *O*_d18_).

2) The second set of variables are BW and backfat of sows, and piglet weight on d 3 and 18 of lactation (*X*_1_, *X*_2_, *X*_3_, *X*_4_, *X*_5_, and *X*_6_).

**Table 6:** Canonical correlations between oxidative stress indicators and reproductive performance of sows in Exp. 2

The first set of variables1)	The second set of variables2)	Canonical correlation coefficient	p-value	Canonical variable composition
Oxidative stress indicators	Piglet weight	0.999	0.024	*V*_1_ = 0.482*M*_d3_ − 0.215*P*_d3_ + 0.823*O*_d3_ *W*_1_ = −0.187*X*_5_ − 0.986*X*_6_
Oxidative stress indicators	Sow litter size	0.999	0.023	*V*_1_ = 0.181*M*_d90_ − 1.029*P*_d90_ + 0.077*O*_d90_ *W*_1_ = 0.257*X*_7_ + 0.889*X*_8_

1) The first set of variables are oxidative stress indicators including concentrations of malondialdehyde, protein carbonyl, and 8-hydroxy-deoxyguanosine on d 90 of gestation, and d 3 of lactation (*M*_d90_, *P*_d90_, *O*_d90_, *M*_d3_, *P*_d3_, and *O*_d3_).

2) The second set of variables are piglet weight on d 3 and 18 of lactation, and litter size of born alive and wean of sows (*X*_5_, *X*_6_, *X*_7_, and *X*_8_).
